# Diamine-Crosslinked and Blended Polyimide Membranes: An Emerging Strategy in Enhancing H_2_/CO_2_ Separation

**DOI:** 10.3390/polym17050615

**Published:** 2025-02-25

**Authors:** Noor Hafizah Mohd Amin, Mohd Usman Mohd Junaidi, Zulhelmi Amir, Nur Awanis Hashim, Hanee Farzana Hizaddin, Abdul Latif Ahmad, Mohd Izzudin Izzat Zainal Abidin, Mohamad Fairus Rabuni, Sharifah Norsyahindah Syed Nor

**Affiliations:** 1 Department of Chemical Engineering, Faculty of Engineering, Universiti Malaya, Kuala Lumpur 50603, Malaysia; 23072942@siswa.um.edu.my (N.H.M.A.); awanis@um.edu.my (N.A.H.); hanee@um.edu.my (H.F.H.); izzudinizzat@um.edu.my (M.I.I.Z.A.);; 2 Sustainable Process Engineering Center (SPEC), Faculty of Engineering, Universiti Malaya, Kuala Lumpur 50603, Malaysia; 3 School of Chemical Engineering, Universiti Sains Malaysia Engineering Campus, Nibong Tebal 14300, Pulau Pinang, Malaysia; chlatif@usm.my; 4 Department of Biomedical Engineering, Faculty of Engineering, Universiti Malaya, Kuala Lumpur 50603, Malaysia

**Keywords:** hydrogen purification, membrane technology, polymer membrane, polymer blends, crosslinking, membrane modification

## Abstract

The increasing demand for high-purity hydrogen (H_2_) as renewable energy sources is driving advancements in membrane technology, which is essential for achieving efficient gas separation. Polyimide (PI) membranes have become an emerging option for H_2_/CO_2_ separation due to its excellent thermal stability and stability under harsh conditions. However, the neat PI membrane suffers performance loss due to CO_2_ plasticization effect and an encountered trade-off limit between permeability and selectivity. Therefore, membrane modification by crosslinking and blending emerged as a recent strategy to enhance the membrane’s performance and properties. This paper provides: (1) An overview of the possible method to do the modification in PI membranes, including the advantages and challenges of the membrane modification types; (2) As blending and crosslinking is the most popular modification for the PI membrane, their roles in enhancing membrane properties for improved H_2_/CO_2_ separation are discussed; (3) The critical parameters of the blending and crosslinking processes are also clarified for the optimal purification process; (4) The future outlook for H_2_/CO_2_ separation using membrane technology is discussed, aiming to provide commercialization strategy for optimal H_2_/CO_2_ separation. Thus, this review could provide guidelines for the readers to implement changes that significantly enhance the membrane’s features for high-purity H_2_ production.

## 1. Introduction

Global warming, or climate change, is a consequence of greenhouse gas (GHG) emissions. The utilization of fossil fuels to fulfil energy needs has tremendously increased CO_2_ emissions since the combustion of fossil fuels generated carbon dioxide (CO_2_), leading to the main cause of global warming [1,2]. Therefore, there is an urgent call to reduce the GHG released into the environment, highlighting the need for energy transition, with H_2_ energy emerging as a promising solution. H_2_ gas has emerged as a clean energy source that can serve as an alternative to fossil fuels due to its potential to generate electricity through the application of fuel cells [3,4]. H_2_ is acknowledged to reduce the greenhouse effect by not emitting harmful pollutants or GHGs into the environment. This is because H_2_ only produces water (H_2_O) as a by-product with no carbon emissions when used as fuel [5,6]. So, this factor is driving hydrogen demand growth, and it is expected to reach 145 million metric tons in 2030 and 660 million metric tons by 2050 [7].

**Figure 1:**
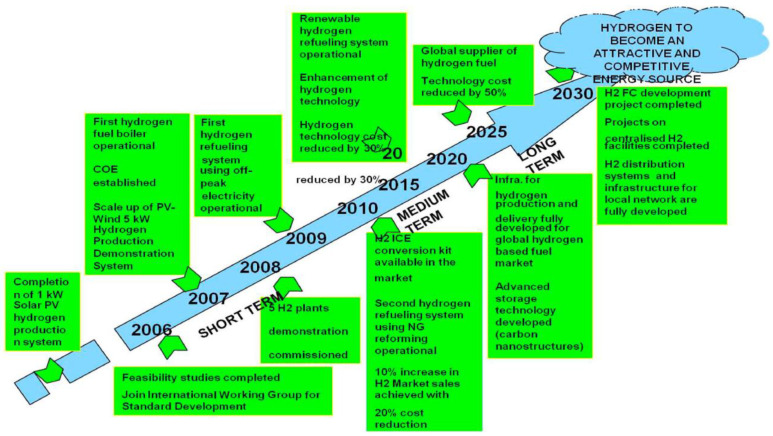
Roadmap for H_2_ energy in Malaysia. Reproduced from [9], Elsevier, 2021.

In Malaysia’s case, Malaysia aims to achieve carbon neutrality by 2050, which aligns with its goal in the 12th Malaysia Plan by lowering GHG emissions, especially by decreasing reliance on fossil fuels and embracing renewable energy sources [8]. Figure 1 depicts the details of the roadmap for H_2_ energy in Malaysia. The roadmap outlines a clear growth plan for hydrogen fuel cells (HFCs) in the short, medium, and long term, with a focus on generating H_2_ from renewable resources starting in 2030 to realize the H_2_ economy [9].

### 1.1. Potential Applications of H_2_

**Figure 2:**
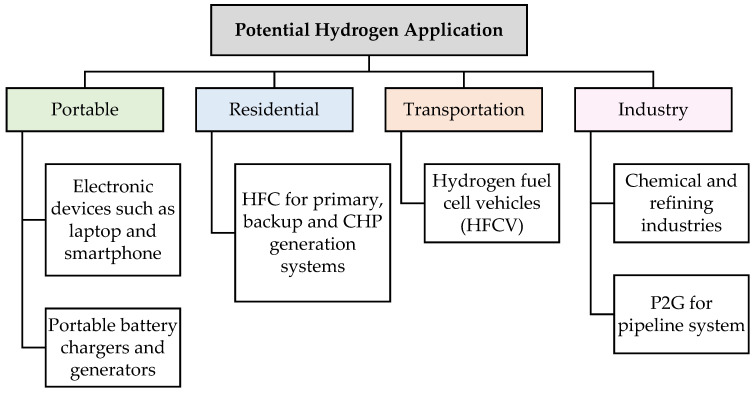
Potential H_2_ application in Malaysia.

In alignment with the objectives outlined in the roadmap, extensive studies on H_2_ production technologies have been explored, including biomass gasification [10,11,12], reforming of bio-liquids [13,14], reforming of biogas [15,16], dark fermentation [17,18,19], photoelectrochemical water splitting [20,21], and microbial electrolysis cells [22,23]. Those technologies can generate H_2_ by utilizing renewable sources such as water, sunlight, and biomass. Additionally, H_2_ has significant potential for various applications in Malaysia, including devices, residential, vehicle, and industrial sectors (Figure 2) [24].

In Malaysia, H_2_ has been explored in the field of HFC technology, showing a variety of benefits. It can be used as portable battery chargers and generators, as well as in electronic devices such as laptops and smartphones, which are cost-effective and have a long lifespan [24]. Furthermore, it serves as an effective solution for residents by functioning as a primary, backup, or combined heat-power (CHP) generation system, making it adaptable to various climates and environmental conditions [24].

Interestingly, H_2_ can also be blended with natural gas to improve combustion efficiency through a process known as Power-to-Gas (P2G) within the natural gas pipeline system [24]. Moreover, H_2_ is also commonly used in the chemical and refining industries. In refineries, H_2_ used to break down heavy residual oils into lighter products, as well as remove impurities from hydrocarbons [25]. In the chemical industry, H_2_ is used for the production of ammonia and fertilizers [26].

In transportation applications, H_2_ serves as a fuel for hydrogen fuel cell vehicles (HFCVs), making it a potential replacement for conventional vehicles [24]. In 2019, Sarawak Energy Bhd opened Southeast Asia’s first integrated H_2_ production and refueling station, and by 2020, three hydrogen-powered buses commenced service [24]. Recent research explored the potential of incorporating a certain amount of H_2_ into the combination of natural gas (NG) and/or liquid petroleum can enhance combustion efficiency and extend the lifespan of internal combustion engines [27,28].

While there are various potential applications for H_2_, achieving high purity is essential for unlocking its full potential in those mentioned applications. By focusing on the H_2_ production quality, H_2_ can be performed optimally and meet the demands of its applications.

### 1.2. H_2_ Purification Technologies

To fully utilize H_2_ as an alternative energy, H_2_ purification from any contaminant gases known as impurities is critical. Acid gases, particularly CO_2_ gas, pose a considerable challenge as major impurities in H_2_ gas streams. Addressing these impurities is crucial for ensuring the purity and efficiency of H_2_ production, which is essential for various industrial applications and the transition to cleaner energy sources. For instance, to utilize H_2_ in fuel cells, high purity of H_2_ is needed (>99.99%) [29]. The presence of CO_2_ at specific concentrations might cause cell poisoning [30] and decrease the operational lifespan of the fuel cell [31].

At present, there are several types of H_2_ purification technologies available, including cryogenic distillation, absorption, adsorption, and membrane technology [32,33,34]. However, three types of these methods (cryogenic distillation, adsorption, and absorption) show more drawbacks than advantages, such as a complicated method to operate, high-cost usage, and high energy usage than membrane technology. Aminullah et al. [35] reported that the system utilizing membrane separation technology has the lowest payback period and is more economical than other purification technologies. Therefore, this simple and cost-effective membrane technology should be an interesting option for investors to apply in their application. The comparison between the H_2_ purification technologies is shown in Table 1.

### 1.3. Membrane Technology

**Figure 3:**
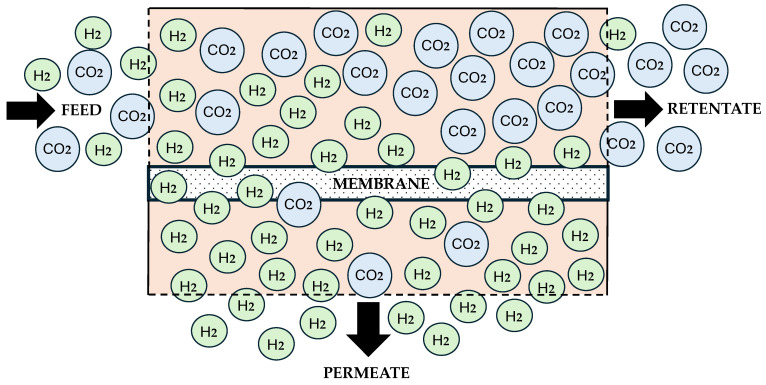
The schematic diagram illustrates the membrane separation configuration used in the crossflow process.

Membrane technology has been the subject of extensive research in the field of H_2_ purification [36,37,38,39] due to its low cost and simple manufacture and processability compared to other H_2_ purification methods. The most challenging part of purifying H_2_ is separating it from CO_2_ gases. During the purification process in the membrane reactor, the feed contains a mixed gas with H_2_ and CO_2_ as major components. The membrane functioned as a thin selective barrier that enables the specific component, which is H_2_, to pass through. This separation system has three key regions: the feed, the retentate, and the permeate (Figure 3). The mixed gas enters the membrane system through the feed region. For the H_2_-selective polymer membrane, it is designed to allow only H_2_ to pass through the permeate region while the other gases remain in the retentate region [40]. The mixed gas molecules will follow the solution-diffusion mechanism [41]. In simple terms, it enables H_2_ gas to permeate through the adsorption and diffusion process. This diffusion is driven by chemical potential differences. Here, the kinetic diameter and condensability of gas molecules will become the critical parameters for the separation process.

The process began with the introduction of a gas mixture of H_2_ and CO_2_ through the feed region. These gas mixtures are then absorbed onto the membrane surface, and the mixture gas diffuses selectively based on a few criteria, such as its kinetic diameter, solubility, and diffusivity properties.

Generally, it is known that H_2_ and CO_2_ molecules have slightly different kinetic diameters; specifically, H_2_ has a smaller size of 2.89 Å, while CO_2_ has a larger size of 3.30 Å [42]. It allows the selective H_2_ gas molecules to permeate from the membrane more effectively than CO_2_ gas molecules, moving into the permeate side. Inversely, the CO_2_ gas molecules that are unselected by the membrane remain on the feed side and exit into the retentate region [40].

It is also speculated that the smaller size of H_2_ gas molecules results in a higher diffusion coefficient property, allowing them to diffuse more quickly through the membrane and desorb into the permeate region [43]. In contrast, CO_2_ gas molecules, which have larger molecular sizes and are more condensable, tend to remain in the membrane, creating a higher concentration in the retentate region [43]. It is worth noting that it is essential to develop strategies that enhance the diffusivity of H_2_ while mitigating the impact of its lower solubility for H_2_ purification. This is because it was reported that CO_2_ molecules are highly condensable in polymer materials [44]. It makes H_2_ compete with CO_2_ gas for the sorption process in the polymer membrane, which leads to lower H_2_ selectivity. Thus, choosing the right membrane material is considerably crucial for successful H_2_ purification and achieving high purity of H_2_.

#### Membrane Materials Section

**Figure 4:**
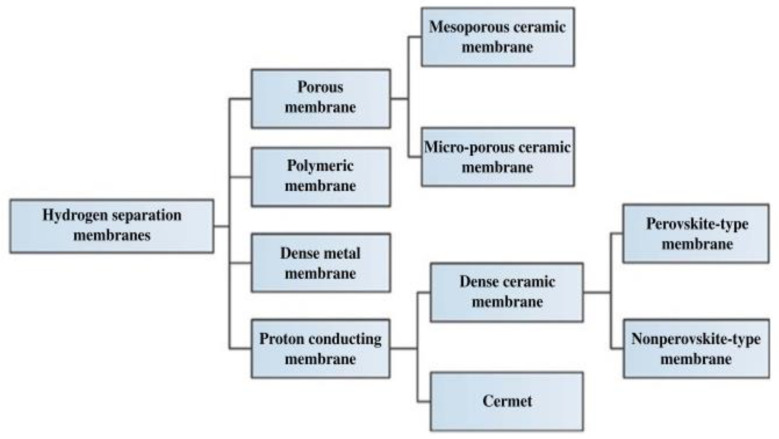
The types of hydrogen separation membranes. Reproduced with permission from [40], Elsevier, 2020.

Membrane material selection for gas separation can be categorized into several types based on their material properties, including porous membranes, polymeric membranes, dense metal membranes, and proton-conducting membranes, as illustrated in Figure 4. The polymer membrane is the only type of organic membrane, whereas all other membranes are inorganic membranes [40]. The porous and proton-conducting membranes are made up of ceramic materials, while the dense metal membranes are composed of palladium (Pd) and its alloys [37]. Porous membranes are classified based on their pore sizes. Microporous ceramic membranes have much smaller pore sizes (<2 nm) compared to mesoporous ceramic membranes, which have pore sizes ranging from 2 to 50 nm [45]. However, porous membranes exhibit low to moderate H2 selectivity when compared to polymer and dense metal membranes [40].

Proton-conducting membranes can be categorized into two types: dense ceramic and composite ceramic metal (cermet) materials. Dense ceramic membranes capable of producing extremely high purity of H_2_ stream at a high temperature (1173 K) [45]. However, it requires high operating temperatures of 700–1000 °C [40].

Dense metal membranes are non-porous. For instance, Pd-Ag alloy-based membranes have proven to produce high-purity H2 (~99.99%) [46]. However, they are responsive to poisoning, such as carbon monoxide (CO) and hydrogen sulfide (H_2_S), which reduce the membrane’s separation performance [47]. In addition, the fabrication cost is also expensive [47]. Thus, ceramic membranes are preferable over dense metal membranes, largely because of their remarkable inertness when dealing with toxic gases [47].

Polymer membranes can be divided into glassy and rubbery membranes. Glassy membranes are made below the glass transition temperature, while rubbery membranes are made above the glass transition temperature [48]. Among the types of polymer membranes, which are glassy and rubbery, the glassy polymer membrane is preferable due to its advantages that have a higher solubility coefficient than a rubbery polymer membrane [49]. In addition, glassy membranes have high selectivity and low flux, whereas rubbery membranes have high flux but lower selectivity [48]. Thus, it makes glassy polymer materials dominate the positions on or near the upper bound in the Robeson plot than rubbery membranes [49].

Furthermore, polymer membranes offer several advantages, such as using lower energy consumption, low fabricated cost, simple operation, and ease of integrating with other technologies for improved separation [36]. However, neat polymer membranes easily swell due to the CO_2_ plasticization effect [50]. Not only that, there is a performance trade-off limit between the permeability and selectivity as indicated in the Robeson plot, which makes the membrane either have high permeability but low selectivity, and vice versa [51].

After considering the advantages and disadvantages of the various membrane material types discussed above, polymer membrane technology emerges as the most preferable option for H_2_ purification. Polymer membranes use a simple and cost-effective fabrication method and consume less energy than inorganic membranes.

### 1.4. Polymer Membranes for H_2_/CO_2_ Separation

Many types of polymer materials have been introduced to separate the H_2_ from the CO_2_ gas, including PI, polysulfone (PSf), acetate cellulose (AC), and polycarbonate (PC) [52]. However, as mentioned before, these neat polymer materials showed poor separation performance either in selectivity or permeability for the H_2_/CO_2_ application. Teplyakov [52] reported that H_2_/CO_2_ selectivity for those polymer materials did not even exceed 8; it varies between 1.7–7.6 in the laboratory scale. It is even worse for industrial polymer materials with H_2_/CO_2_ selectivity up to 3.8 only, as indicated in Table 2.

It should be noted that the best membrane features for gas separation are evaluated from two key parameters, which are the permeability of the desired gases and the separation factor known as selectivity [51]. The major challenge that membrane technology has faced until today is the trade-off limit between this permeability and selectivity. The selectivity of the membrane materials commonly decreases with increasing gas permeability, and vice versa [51]. Moreover, in the field of H_2_/CO_2_ separation, it has indeed remained a challenge to separate and purify H_2_ from the CO_2_ gas efficiently due to the similar molecular kinetic diameters between H_2_ (2.89 Å) and CO_2_ (3.30 Å) [53]. Moreover, as discussed earlier, CO_2_ molecules are highly condensable in polymer materials [44]. It makes H_2_ compete with CO_2_ gas for the sorption process in the polymer membrane, which leads to lower H_2_ selectivity.

In terms of selectivity, it is known that the gas performance depends on the product of solubility selectivity and diffusivity selectivity, known as perm-selectivity [54]. Hu et al. [55] stated that there are few properties affecting solubility selectivity and diffusivity selectivity. Solubility selectivity depends on the gas condensability, interaction between gas molecules and polymer, and also fractional free volume (FFV) [55]. While diffusivity selectivity depends on the gas molecular size, interchain distance in the membrane, and polymer chain flexibility [55]. Low H_2_ condensability and H_2_ diffusivity in the polymer membranes affect the overall perm-selectivity of the membranes [55]. For this reason, modification in the neat polymer membrane was introduced to overcome the aforementioned issue.

### 1.5. PI Membranes for H_2_/CO_2_ Separation

Up until now, PI has been proven as one of the emerging polymer materials for H_2_/CO_2_ separation. The majority of the discussion in the literature focuses on how PI membranes contribute to enhancing H_2_/CO_2_ separation. Neat membranes made from PI polymer have been used extensively since the late 1990s in H_2_ purification systems due to their outstanding thermal stability and chemical resistance rather than other polymer material types [56]. Undoubtedly, PI membranes stand out as the predominant choice for H_2_ purification by far when compared with other glassy polymer materials used for H_2_/CO_2_ separation, as indicated by Yanez et al. [57].

Generally, PI is a polymer that contains an imide group in its chemical structure. PI has high glass transition temperature, T_g_ (>300 °C), and high stability (>400 °C) [40]. Several types of PI membranes have been developed for H_2_/CO_2_ separation, such as 6FDA-durene, Matrimid P84, and PMDA, and the structures of the PI membrane have been reviewed extensively in other literature elsewhere [58]. However, despite its benefits, PI commercial membrane suffers limitations due to the CO_2_ plasticization effect. This significantly shows that PI is unfavorable with a high CO_2_ concentration feed stream [59,60]. Commonly, the PI membrane will swell and change its properties, especially the free volume and inter-chain spacing, which then reduce the H_2_ selectivity. For this reason, surface modification on the polymer membrane’s matrix has been introduced to overcome the limitation that PI suffers by blending the PI material with other polymers during the preparation stage [61,62,63] and crosslinking the PI chain with a suitable crosslinker [44,64,65].

It has been reported that blending these PI materials with two or more polymer materials significantly improved the membrane durability [53,61]. For instance, the presence of PES and PSf materials significantly improves the modified membrane’s tolerance to plasticizing gases, CO_2_ gas at high pressure [61,62]. In addition, H_2_ selectivity can be improved by crosslinking the polymer backbone with the diamines, as reported by many researchers [44,53,65]. Many factors need to be considered for optimal H_2_ purity, especially the synthesis method and crosslinking parameter condition. This will be discussed in Section 3.2.

Thus, this review provides an overview of the membrane modification in PI membranes. The possible method to modify PI membranes is discussed, as well as the advantages and challenges of the membrane modification types. As blending and crosslinking are the most popular modifications for PI membranes, their role in modifying the membrane’s properties for enhancing H_2_/CO_2_ separation is also discussed. In addition, the critical parameter condition of the blending and crosslinking processes for the modification in PI membranes is also clarified because it is essential for optimizing their performance.

**Figure 5:**
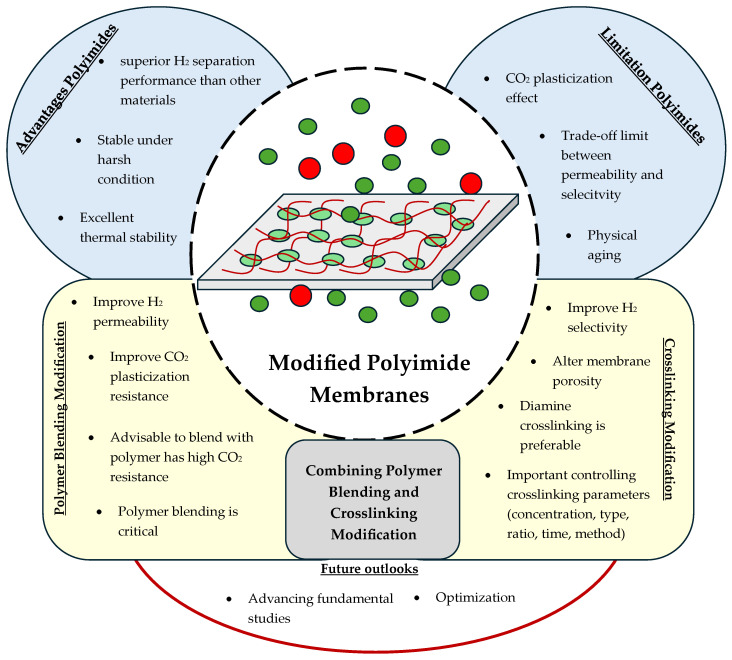
A schematic diagram illustrating the modification of the PI membrane for H_2_/CO_2_ separation discussed in this paper.

A thorough understanding of these parameters will help the readers to implement effective modifications that significantly enhance the membrane’s features. Consequently, this knowledge will lead to superior efficiency in H_2_/CO_2_ separation, ensuring that the membranes deliver optimal results in this important application. Figure 5 summarizes the overview of the contents of this paper.

## 2. Membrane Modification Strategy

To overcome PI’s limitation for H_2_/CO_2_ separation, membrane modifications, specifically on the membrane surface, are initiated. Many research efforts have been explored to improve H_2_/CO_2_ separation efficiency. The research focus is to engineer neat PI membranes, with the aim of improving molecular chain packing by creating more narrowly distributed free volumes [55]. Therefore, this section provides an overview of membrane modification in PI membranes, including the advantages and challenges of the modification methods.

The strategies for membrane modification can be categorized into two main types: physical modifications and chemical modifications. Physical modifications focus on modifying the structures and properties of the membrane without changing their chemical composition. Physical modifications typically involve blending [61,63,66] or adding additives [55]. Chemical crosslinking is preferable to other chemical modification methods for PI membranes. As discussed before, membrane modification is a strategy to break through the permeability and selectivity trade-off limit on the Robeson upper-bound plot by improving selectivity, permeability, and overall efficiency.

When reviewing the aforementioned strategies, most researchers prefer to use diamines as crosslinkers in the crosslinking process to modify PI membranes or blend them with PSf or PES materials for H_2_ enrichment. Moreover, researchers chose advanced strategies to combine both approaches to enhance H_2_ purification. The details for the modifications were discussed in Section 5.

### 2.1. Advantages and Disadvantages of Diamine Crosslinking Modification

Crosslinking modification has emerged as an emerging membrane modification for PI membranes by achieving higher gas perm-selectivity [67]. This modification can resist the PI membrane swelling that commonly occurs due to CO_2_ plasticization. It is known that CO_2_ plasticization affects the membrane performance by increasing the gas permeability and decreasing the preferred gas selectivity. By crosslinking, the permselectivity for the PI membranes improved by maintaining the high permeability and increasing the membrane resistance against the aggressive gases [67]. Not only that, experimental findings from Zhang et al. [68] indicated that crosslinked PI membranes showed good long-term stability over 1100 h when compared with non-crosslinked PI membranes.

Inspiring by these advantages, extensive research has been developed for modifying the neat PI membranes, including incorporation of the crosslinkable group in the PI synthesis, introduction of diamine crosslinking through carbonyl reaction, and synthesis of branched PI [69,70]. However, custom synthesis is not preferable for the modification method of the PI membrane because it is tedious work that consumes time and is not applicable to large-scale applications [70], making crosslinking preferable for the modification.

In addition, diamine crosslinking is proven to reduce the physical aging of the membrane films by a simple immersion step of the PI membrane films in crosslinker methanol solution conducted by Zhou et al. [71]. The crosslinked membrane PI films with a thickness of 1.5 μm suppress the physical aging, which is less than the 15% loss of permeability after about 35 h of the heat treatment [71]. This is because the crosslinking tightened the interstitial space between polymer chains, resulting in the restriction of the polymer chain from being flexible, and, as a result, it enhanced the O_2_/N_2_ from 3.3 to 4.5 [71].

Despite these advantages, crosslinked PI membranes also encountered main advantages: gas permeability loss is commonly observed. The study by Nasir et al. [64] demonstrated that while H_2_/CO_2_ selectivity is enhanced by PDA-crosslinking modification, the H_2_ permeability for the crosslinked PI membranes decreased dramatically with higher PDA concentration in the crosslinking solution. The decrease in H_2_ permeability may limit the overall efficiency of the gas separation process. This is because a membrane with high gas permeance is desirable for high productivity and lower capital costs [64]. Due to the aforementioned issue, this factor must be carefully considered to optimize the PI membranes’ performance for H_2_ purification.

### 2.2. Polymer Blending for PI Membranes

Research studies have been focused on blending two polymers’ materials by considering that a single polymer does not produce satisfactory performance in purifying interested gas. This blending modification offers benefits in terms of modifying the existing polymer without the need to undergo the whole process of the new polymer synthesis [72]. Moreover, this blending modification combines the advantages of each polymer’s properties into a new targeted membrane features [73]. Depending on the optimal composition of the blend, improved performance in permeability and selectivity could be achieved, overcoming the deficiencies in each polymer’s properties.

As evidenced by the work of Hamid et al. [61], the PSf/PI blends membranes with an equal ratio of each polymer, achieving higher H_2_/CO_2_ selectivity and increasing from 3.1 (neat PI membrane) to 4.4 (PSf/PI blend membrane). In addition, the blend membrane was proven to resist the CO_2_ plasticization effect at the pressure of the 5 bar with a duration of 360 min [61], and a previous study done by Kapantaidakis et al. [74] also proved that this polymer blend membrane is able to withstand CO_2_ plasticization up to 30 bar.

It is notable that neat PI membranes commonly encountered CO_2_ plasticization under high pressure as CO_2_ acted as a plasticizer in the mixed gas [59,60]. The evidence of this phenomenon can be observed in the membrane through a noticeable decline in its perm-selectivity. As perm-selectivity decreases, the membrane becomes less effective at distinguishing between different gases, which can result in a higher overall gas permeability. It was reported by Lin and Yavari [75] that, at a high CO_2_ concentration in the feed stream, sorbed CO_2_ swells the polymer matrix, thus reducing the membrane separation performance, especially membrane selectivity. Therefore, PI materials are commonly blended with polymers that have higher CO_2_ plasticization to suppress CO_2_ plasticization. For example, PSf has been introduced as a potential blending polymer by Kapantaidakis et al. [74,76]. They validated good blending compatibility of PSf and PI and achieved compelling anti-plasticizing ability that enhances the CO_2_ plasticization resistance even at high feed pressure (>30 bar).

This polymer blending modification also offers other advantages, including cost and time savings, as well as a simple procedure in the modification steps [73]. Thus, this benefit makes blending modification suitable for PI membranes. However, compatibility in the polymer blends at the molecular level must be considered as it will affect membrane performance [73]. The importance of compatibility in the polymer blend is also reviewed by Fakirov [77], highlighting the importance of compatibility in polymer blends. The differing nature of the components may cause antagonism on the contacting surfaces.

## 3. Diamine-Crosslinked PI Membranes for H_2_ Purification

### 3.1. Crosslinking Strategy

**Figure 6:**
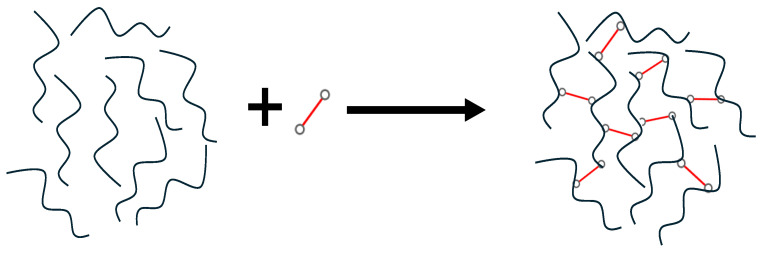
The chemically crosslinked process on polymer chain (black line) by crosslinker (red line) reduces free volume in the membrane system.

Crosslinking is one of the most well-known surface membrane modifications for PI membranes to regulate and control the interstitial d-spacing and chain stiffness in the polymer network [78]. These two parameters are very crucial in determining their gas permeation properties as propounded by Freeman’s theory [54]. Chemical crosslinking is extensively studied by researchers to enhance the PI membranes by improving their perm-selectivity and plasticization resistance, as reported by [70,79,80,81]. This crosslinking type is a bond formation that links the functional group in the polymer chain to another polymer chain in the form of a covalent bond, forming a three-dimensional network (Figure 6). The covalent bond is the most stable bond and the most preferable for PI membranes [82].

Generally, the formation of the covalent bond will restrict the chain’s ability to move or be less flexible). It thus provides not only good chemical resistance and anti-plasticization but also better long-term performance stability. Other than that, this crosslinked formation will indirectly alter the polymer structure by increasing or decreasing the membrane’s porosity, which affects the molecules’ diffusivity into the polymer network. Depending on the type of crosslinker, the crosslinker may contain two, three, or more crosslinking sites. The following subtopics will discuss further details on the effect of crosslinking parameters.

### 3.2. Diamine-Crosslinked PI Membranes

Up to the present, diamine has been widely used as a crosslinker for crosslinking modification in PI membranes. It was first introduced by R.A. Hayes [83] for gas separation and progressively studied by Chung’s group [44]. In the study, PDA-crosslinked PI (6FDA-durene) membranes have been discovered to increase the H_2_/CO_2_ selectivity up to 101 for the pure gas test and 42 for the mixed gas test. Three types of linear crosslinkers have been used: ethylenediamine (EDA), 1,3-diaminopropane (PDA), and 1,4-diaminobutane (BuDA). The decrease in mixed gas is due to the sorption competition between H_2_ and highly condensable CO_2_ gas [44]. The study also highlights that the effectiveness of the crosslinker may be due to the molecular length of crosslinker molecules, which allows sufficient crosslinking reactions to occur. Despite all three crosslinker types having similar widths (3 Å), their molecular lengths are different, which influences the diffusion of the crosslinker into the polymer and, thus, affects the reaction rates [44]. Short crosslinkers may easily diffuse into the membrane, while longer crosslinkers reduce the potential of crosslinking reaction due to limited potential to diffuse into the polymer [44].

By using the same linear crosslinkers (EDA, PDA, and BuDA), Low et al. [84] modify another type of PI membrane, which is 6FDA-ODA/NDA. The study also shows that PDA-crosslinked PI membranes improve results by increasing the H_2_/CO_2_ permselectivity of the pure gas test from 2.3 (neat PI) to 64 (PDA-crosslinked PI) for 90 min of immersion time. The mixed gas test shows the same trend as discovered by Chung et al. [44], where it decreases the H_2_/CO_2_ permselectivity to 45. The study also highlights the importance of considering the appropriate selection of crosslinker and crosslinking reaction time to crosslink the polymer chain while maintaining the main chain rigidity [84].

**Figure 7:**
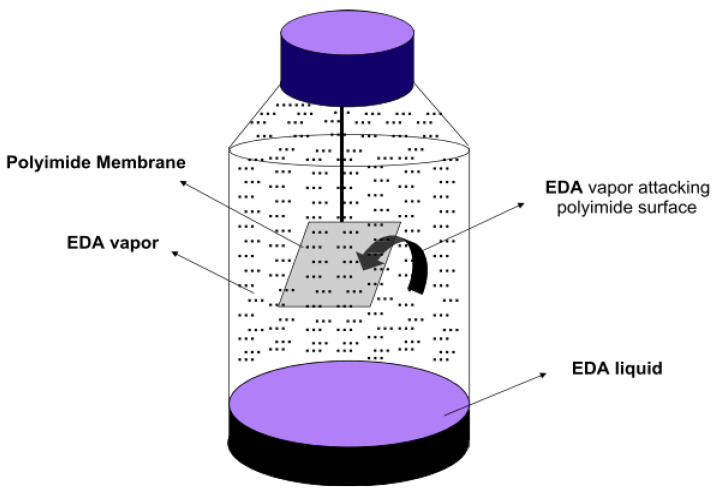
The schematic diagram of the vapor phase method proposed by Shao et al. [85]. Reproduced with permission from [85], Elsevier, 2009.

Shao et al. [85], in another study, used the same crosslinker, EDA, to crosslink the same PI membrane types with a different modification method, which used the vapor-phase method. As illustrated in Figure 7, the modification was performed in a home-made container, where the membrane is quickly exposed to the vapor phase of the EDA solution without any contact, with the aim to only modify the physicochemical properties of the outer PI membrane without modifying the internal membrane structure [85]. The results show that the H_2_/CO_2_ selectivity increased up to ~100 (pure gas test) under only 10 min vapor crosslinking modification, discovering a new crosslinking modification method for PI membrane with a simple method and efficient time and cost, which is applicable for large-scale processes.

In another study by Shao et al. [86], the PI membrane performance is evaluated by crosslinking the PI membrane with different generations of diaminobutane (DAB) dendrimer. It was found that modified 6FDA-durene films with DAB-AM-4 (G1) dendrimer at 35 °C for 60 min enhance the H_2_/CO_2_ selectivity up to 265%. The studies unravel the efficiency of smaller crosslinker molecules in improving the gas performance by tightening the polymer chain, filling the space, and rearranging the free-volume distribution [86].

Nasir et al. [65] discovered the effectiveness of crosslinking modification by immersing the PI (P84) membranes into the BuDA crosslinker in the methanol solution. Surprisingly, the findings show that the crosslinked BuDA has a significant impact on the H_2_ permeability of P84 membranes [65]. The H_2_ permeability performance of the PI membrane increased up to 804.1 (320.59% increases) with BuDA in 10 min crosslinking immersion time, and then H_2_ permeability decreased to 602.7 using the BuDA crosslinker in 30 min of crosslinking immersion time [65]. In the 10 min crosslinking time, the attractive hydrogen bond between BuDA and H_2_ is dominant, resulting in increasing the H_2_ permeability and decreasing the H_2_/CO_2_ selectivity from 5.31 (neat PI) to 3.90 (crosslinked PI). Inversely, the H_2_/CO_2_ selectivity increased to 6.09 using BuDA in 30 min of immersion time [65]. However, in 30 min of immersion time, H_2_ permeability decreases slightly in about ~15%, and the H_2_/CO_2_ selectivity increases up to 6.09 (crosslinked PI) [65]. This is because BuDA is sufficiently crosslinked with the polymer chain, so the crosslinking network is tighter, resulting in a decrease in interstitial space of the polymer chain for a longer time of crosslinking reaction [65].

Another study by Nasir et al. [64] demonstrated that the mixing of two different types of linear diamine, PDA–BuDA, was able to increase H_2_ selectivity up to 7.84 from 3.7 (neat PI) by using the PI membrane with an 8:2 ratio of PDA:BuDA. In comparison with the research conducted by this group in the previous year [65], it can be seen that mixing these two types of crosslinkers significantly increases H_2_ selectivity more than using a single crosslinker. The exploration of this new strategy might be considered if it is suitable to be used in large-scale processes by considering the cost efficiency and simplicity of the process.

Choi et al. [87] discovered the potential of crosslinking the 1,5-diamino-2-methylpentane (DAMP) with PI hollow fiber membranes for H_2_/CO_2_ separation. The findings proposed that crosslinking the PI membrane with 10 wt. % of DAMP enhances H_2_/CO_2_ selectivity up to 16.1 from 5.3 (neat PI) and decreases H_2_ permeability [87], as expected in the previous studies. Surprisingly, the membrane performance is somewhat higher than the slope in the Robeson upper bound highlighting the effectiveness of the crosslinking modification for the PI hollow fiber membrane [87].

López–Badillo et al. [88] crosslinked the PI (6FDA-6FPA) membrane using 1,5-pentadiamine (DAP) in a methanol solution by varying the crosslinker concentration. The result shows that the H_2_/CO_2_ perm-selectivity increases up to 7.44 using 3% *v*/*v* of DAP under 120 min of immersion time, and it was located near the upper limit in the Robeson plot, highlighting the influence of FFV in selectivity enhancement [88].

Table 3 compares H_2_/CO_2_ selectivity between unmodified and modified PI membranes. Amines exhibit high nucleophilicity, which leads to a reaction that may occur at room temperature or without the presence of a catalyst. This strategy is cost-effective and uses the simplest modification method despite showing a significant enhancement in H_2_/CO_2_ perm-selectivity, as mentioned earlier.

### 3.3. Impact of Crosslinking Parameters on Membrane Properties and Performance

Although crosslinking modification can significantly enhance membrane performance, various factors must be considered to maximize H_2_ purification from the biogas mixture. Reaction parameters of crosslinking reactions are very important in enhancing the membrane’s properties for better H_2_ separation. H_2_ purification is significantly influenced by process parameters like crosslinking time, crosslinker types and concentration, modification methods, and many more. Most of the time, the purpose of the crosslinking reaction in the membrane system is to maximize separation performance. Hence, it is important to discuss the effect of these process conditions on H_2_ purification using these crosslinked PI membranes.

#### 3.3.1. Effect of Crosslinking Time Correlated with Molecular Structures

The permeability and selectivity of the H_2_ gas into the PI membrane significantly correlated with the immersion time of the membrane into the crosslinker solutions. Generally, it affects the diffusion rate at which the crosslinker passes through the polymer chain network and forms the bonding between the polymer chain [64]. Thus, crosslinking time plays an important role in influencing the polymer chain configuration and altering the free volume of the membrane structure [53]. Increasing the crosslinking time reduces membrane-free volume due to the increase in crosslinking density and the tightening of the interstitial spacing between polymer chains. For this reason, H_2_ selectivity increases because when the crosslinker starts to crosslink with the polymer chain, it tightens the interstitial spacing, reducing the FFV. Inversely, the permeability of H_2_ gas decreases [89]. The same trend also shows for different separation of gases [90].

**Figure 8:**
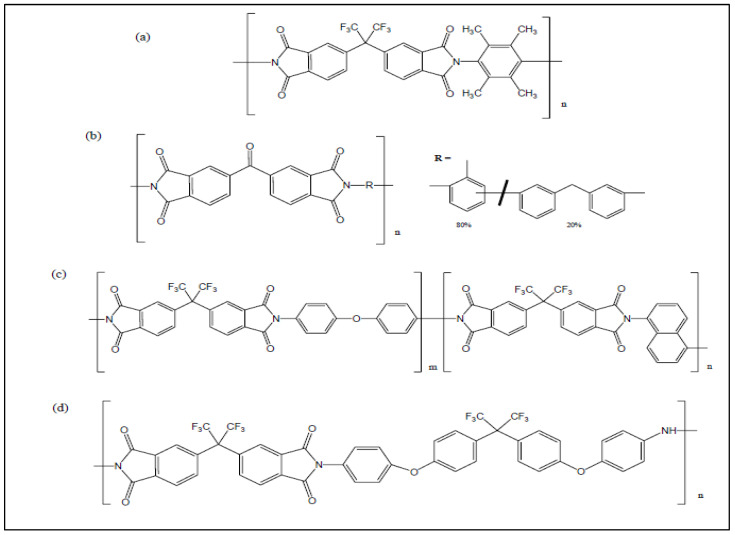
The different types of PI. (**a**) 6FDA-durene; (**b**) P84; (**c**) 6FDA-ODA/NDA; and (**d**) 6FDA-6FPA (created molecular structures using ChemDraw Pro 8.0).

Referring to Table 3 and Figure 8, it can also be noticed that the complexity of the PI types and crosslinker structures contributed to the effectiveness of the crosslinking reaction. Both polymer main chains and side chains are attributed to the crosslinking rate reaction. By comparing the PI types with the same immersion time and crosslinker types (using a PDA crosslinker with 5–15 min immersion time), it can be seen that P84 shows the best performance of H_2_/CO_2_ selectivity with 7.3 in 5 min, followed by 6FDA-durene with approximately 40 in 5 min, 6FDA-ODA/NDA with 6.6 in 15 min (Refer Table 3). Regarding H_2_ permeability, P84 significantly shows the best performance when compared with the other two PI types. It can be seen that there is no bulky trifluoromethyl (CF_3_) in the side chain of P84, which makes the PDA crosslinker diffuse into the polymer chain and crosslink the polymer chain faster. Thus, bonding with polymer chains is efficiently formed in a short time, resulting in better performance using the crosslinked membrane and allowing only H_2_ molecules to permeate better than other PI types. The same hypothesis can also be seen even if EDA is used for the crosslinking reaction (Refer to Table 3).

It should be noted that increasing crosslinking time optimally helps the crosslinker to crosslink the polymer chain in sufficient time to react. However, despite its importance, limited studies have focused on identifying the optimal crosslinking time. Determining this optimal parameter is essential to minimize both cost and processing time for big-scale processes. Determining this optimal parameter is essential to minimize both the cost and processing time for industrial applications. Other than that, it has been observed that immersion time is not the only factor that influences the permeability and selectivity of the PI membrane, but the structure of the PI membrane also contributes to the membrane performance, making it very challenging to provide a clear idea about the roles of crosslinking time on the enhancement of H_2_ purification.

#### 3.3.2. Effect of Crosslinker Types

Material selection is a critical initial step in experimental design, including choosing the right crosslinking agent for the desired gas separation. Choosing an appropriate crosslinking agent is essential for achieving improved results and optimal performance. This is due to the fact that the properties and strength of the crosslinker are closely correlated to its structures, which determines how it interacts with the polymer chains. It is worth noting that every crosslinking agent possesses their own nucleophilicities and molecular dimensions, which affect the efficiency of the crosslinking reaction [84]. While crosslinking typically decreases the polymer chain mobility, the resulting bond formation is dependent on the nucleophilicity and molecular size of the crosslinker [84].

**Figure 9:**
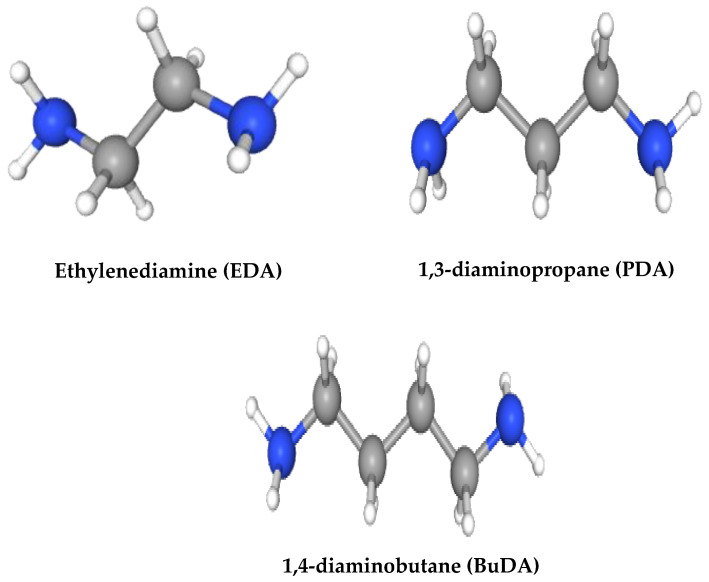
The molecular structures of EDA, PDA, and BuDA (created using TmoleX 4.0).

Low et al. [84] investigated the effect of crosslinker types (EDA, BuDA, and PDA) on H_2_/CO_2_ separation performance. The appropriate nucleophilicity and moderate molecular dimensions of the PDA crosslinker provide the highest degree of crosslinking for the 6FDA-ODA/NDA membrane with an ideal H_2_/CO_2_ perm-selectivity of 64. BuDA, with its largest molecular dimension and lowest nucleophilicity, faces difficulty in penetrating the polymer matrix. As a result, the crosslinking reaction primarily occurs on the membrane surface and is less effective due to chemical grafting and etching, which fail to effectively hinder the chain-to-chain movement. A similar effect was observed by Nasir et al. [64], where the mixture ratio of PDA:BuDA with a high content of PDA presented the highest H_2_ selectivity due to the fact that the molecular length of the crosslinker type influences gas penetration. More detailed effects on the crosslinker concentration were discussed in the later subtopic. Figure 9 shows the molecular structure of EDA, PDA, and BuDA, and Table 4 shows the comparison characteristics between BuDA, EDA, and PDA.

Other research studies also emphasized that the molecular size and reactivity of the crosslinker are the key factors influencing membrane performance. Shao et al. [86] also emphasized that the molecular size and reactivity of the crosslinker are the key factors influencing membrane performance. They investigated three generations of diaminobutane (DAB) dendrimers with different molecular sizes: DAB-AM-4 (G1), DAB-AM-8 (G2), and DAB-AM-16 (G3), which can crosslink PI membranes at room temperature. They concluded that G1 demonstrates the highest selectivity, achieving an impressive increase of up to 265% in H_2_/CO_2_ separation. The best performance of G1 is attributed to its smaller molecular size compared to G2 and G3, which have larger molecular sizes. Since G1 has the smallest size compared to G2 and G3, it achieves the highest degree of crosslinking, which leads to better reactivity towards the crosslinking reaction. As a result, G1 shows the highest performance in H_2_/CO_2_ separation when compared to the PI membrane that crosslinked with G2 and G3.

From this, it can be seen that the different molecular size of the crosslinker used for the crosslinking reaction produces different permeability and selectivity of the gas separation because the molecular size of the crosslinker controls the diffusion coefficient in the polymer matrix of the PI membrane. In addition, the study also emphasized that a crosslinker with a large molecular size significantly causes steric hindrance, preventing the -NH_2_ groups in the dendrimers from completely reacting with polyimides due to the complex structures of both the crosslinker and the polymer chain of the membrane. This also leads to a slower crosslinking reaction in the membrane system, which demonstrates the decrease in the crosslinking degree of crosslinking and reduced crosslinking efficiency [86]. These findings suggested that the role of selecting an appropriate crosslinker in enhancing the membrane properties is critical for better H_2_ purification before evaluating other parameters of the crosslinking reaction.

#### 3.3.3. Effect of Crosslinker Concentration

**Figure 10:**
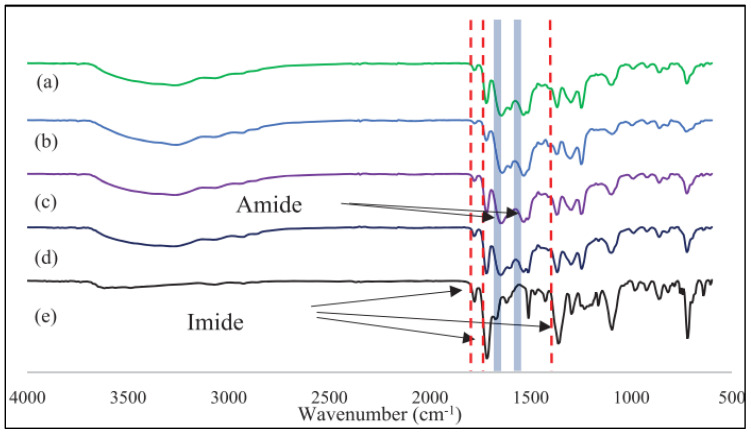
FTIR spectra of original PI membrane and crosslinked membranes. (**a**) 100% PDA; (**b**) 0.8 PDA:0.2 BuDA; (**c**) 0.4 PDA:0.6 BuDA; (**d**) 100% BuDA; and (**e**) pure PI. Imide groups detected from peak of 1778.02 cm^−1^ (C=O asymmetric stretch), 1717.15 cm^−1^ (C=O symmetric stretch and 1366.29 cm^−1^ (C-N), while amide group detected from peak of 1652.94 (C=O stretch) and 1528.62 cm^−1^ (N-H bend). Reproduced with permission from [64], John Wiley and Sons, 2020.

Determining the optimal crosslinker concentration for the crosslinking process is crucial for enhancing selectivity and permeability. Crosslinking significantly increases the rigidity of the polymer chain, affecting both the permeability and selectivity of the gases. Nasir et al. [64] examined the effects of the diamine mixture ratio of crosslinker concentration (PDA: BuDA) on PI membranes for H_2_/CO_2_ separation. It showed that the H_2_/CO_2_ selectivity improved from 3.7 to 7.8 after the modification with a membrane with an 8:2 ratio of PDA: BuDA crosslinker concentration compared to other ratios of the PDA-BuDA solution [64]. Their finding indicated that the degree of crosslinking reactions was influenced by the concentration ratios of the crosslinking agents [64]. Higher PDA concentrations exhibit a higher degree of crosslinking by creating closer chain packing, which then induces a faster crosslinking rate and increases the H_2_ selectivity with higher PDA concentration. Interestingly, the FTIR analysis also shows that the imide group presence is lower with higher crosslinking concentration (Figure 10), indicating the successful modification and interaction between the polymer chain and the crosslinking agents. It is important to balance the degree of the crosslinking rate and the restriction on gas diffusion to achieve optimal performance. This is because 100% PDA concentration potentially swells the polymer chains due to the hydrophilic nature of diamine, which lowers the separation performance in terms of selectivity [64,91].

In another study by Badillo et al. [88], the 6FDA-6FPA PI membrane was crosslinked using 1,5-pentadiamine (DAP) at different crosslinker concentrations (3, 10, and 17% *v*/*v*) and different crosslinking immersion times (10, 65, and 120 min). They speculated that the crosslinking degree is disproportionate to the crosslinking parameters, concentration, and immersion time. This is because optimal H_2_/CO_2_ selectivity was observed with a DPA concentration of 3% *v/v* and an immersion time of 120 min. These findings show the important role of optimizing the crosslinker concentration for optimal membrane performance.

Moreover, it must be noted that excessive crosslinking may lead to an overly rigid network, which limits the practical application in purifying the H_2_ gas, whereas maintaining sufficient flexibility is essential for effectively increasing H_2_ yield in a shorter time. For instance, a highly networked polymer matrix increases the selected gas selectivity due to the crosslinking process. However, it reduced the permeability of the gas, influencing the rigidity of the network for the diffusion of the gas [88]. In addition, the high number of molecules of PDA makes the T_g_ of the polymer decreases from 321° (neat PI) to 315 °C (1 h), 295 °C (6 h), and 260 °C (48 h) because PDA becomes progressively more soluble within the polymer matrix, reacting with a single-chain polymer and forming dangling ends instead of forming the desired interchain bridges [92]. Thus, it is crucial to optimize the crosslinking degree to ensure a balance between selectivity and permeability for effective gas separation, even if the crosslinking degree is indirectly related to low permeability. An equilibrium between the crosslinking degree and the mechanical and transport properties of the membrane is desirable, including the crosslinker concentration [88].

#### 3.3.4. Effect of Crosslinking Modification Method

**Figure 11:**
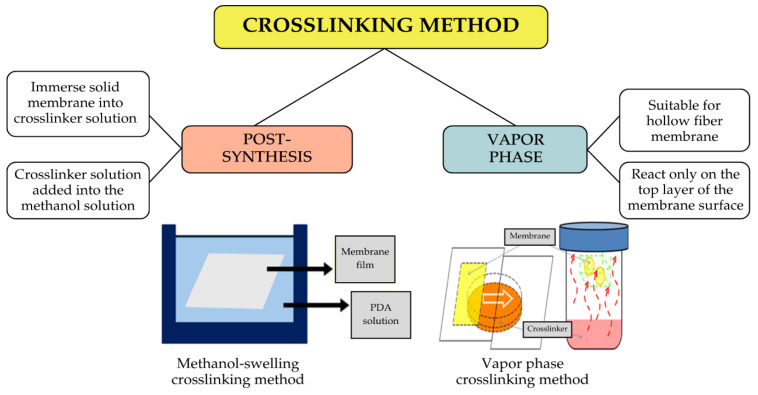
Developed crosslinking method for modifying PI membrane for H_2_ purification In the post-synthesis method, membrane film is immersed in PDA (crosslinker solution), while in vapor phase modification, the membrane (yellow color) is exposed and reacts with the vapor form of amine (pink-orange color). The blue-capped container shows how amine vapor reacts with the membrane surface. Adapted from [93], IOP Publishing Ltd., 2021.

While material selection and its optimal conditions play a crucial role, the crosslinking modification method is equally important, particularly in the context of controlling the coating thickness and better interaction between the crosslinker and the membrane. Within the realm of H_2_ purification, several methods are employed to modify the membrane surface for optimal H_2_ purification, as shown in Figure 11. The methods discussed in this section focus only on the chemical crosslinking method, as it is the most emerging crosslinking method for the PI membrane.

The post-synthesis method is widely recognized as the most employed approach. Liu et al. [94] first discovered the diamine crosslinking method using this post-synthesis. This approach was later extensively investigated by Shao et al. [86], focusing on its potential for H_2_ purification. In the post-synthesis method, PI membrane films, which were first solidified, were immersed in the diamine solution. The diamine solution is prepared in methanol, which serves as a swelling agent for the PI membrane, enabling better access for the diamine molecules to interact with and crosslink the polymer chains within the membrane matrix. The swelling effect is a requisite for promoting effective crosslinking between the crosslinker and the PI structure [86,95].

A similar effect is also observed in the study by Chung et al. [44]. The membrane swelling facilitates the diffusion of the small crosslinker structure across the membrane. However, it is more challenging for the bulky crosslinker structure to penetrate the polymer chains effectively due to the fact that it reduces the d-spacing polymer structure [91]. In addition, this methanol-swelling method has been observed to cause structural changes in the asymmetric membrane, which is undesirable in gas purification. Some of the changes, including an uneven top layer of the membrane surface and variation in pore size, lead to undesirable membrane morphology that can negatively impact the purification process, especially for hollow fiber PI membranes.

Shao et al. [85] then introduced a vapor phase crosslinking modification method to alter the outer layer of the membrane matrix with the aim of maintaining its internal structure. The result indicated that the 10 min vapor phase of EDA treatment significantly improved H_2_/CO_2_ selectivity up to 102. Furthermore, this vapor phase method effectively reduces membrane swelling and enhances the membrane tensile strength with the advantages of this environmentally friendly method as it reduced the chemical used for the modification. However, it is sensitive to humidity and difficult to control the temperature used for the modification [96]. Table 5 shows the summaries of both methods.

#### 3.3.5. Effect of Crosslinker Solution

The crosslinker solution is also one of the parameters to be considered as it directly or indirectly influences the membrane properties and performance. The choice of solvent in the PI membrane modification process may affect the membrane performance and properties [97]. To the best of our knowledge, methanol is the most popular crosslinker solution for the H_2_/CO_2_ separation. The methanol is used to swell the PI membrane film for an effective reaction with the crosslinker [98]. The methanol will swell the membrane, facilitating the diffusion of the crosslinker into the polymer chain and forming a crosslinked structure [98]. Liu et al. [94] also emphasized that the swelling of PI films in the crosslinker methanol solution is necessary for the crosslinking reaction to occur.

Besides that, the effectiveness of the crosslinker methanol solution in the crosslinking reaction depends on the PI types used. Tin et al. [90] found that the crosslinking rate of Matrimid is lower than fluoropolyimides. This is because fluropolyimides have higher free volume and closer solubility parameters to methanol. Therefore, it is easier to swell in methanol than Matrimid [90]. The swelling effect then increases the permeability due to loose chain packing [90]. However, the crosslinking reaction will restrict the mobility of the polymer chain and reduce the FFV. Thus, exploring the effect of crosslinker solution types may be significantly important to improve H_2_/CO_2_ separation.

## 4. Blended PI Membranes for H_2_ Purification

### 4.1. Blended PI Membranes

Numerous studies have demonstrated that the polymer blending strategy significantly offers interesting new membrane features in improving H_2_ purification. Polymer blends represent a unique strategy for creating advanced membranes that exhibit improved permeability performance by leveraging the advantageous properties of individual polymers [99]. Polymer blends can be prepared through melt blending, solution mixing, and others [100]. Generally, polymer blends can be divided based on their phase behavior, which are miscible and phase-separated blends [99]. Miscible blends are formed when two polymers dissolve completely, creating a homogenous solution [99]. While phase-separated blends indicate the combined polymers are partially dissolved and separated by an interface between the two phases [99]. By determining their T_g_, the behavior of the blended polymers can be identified. A single T_g_ indicated that the blend is miscible, while the presence of two different T_g_ indicated that the blend is immiscible [99]. Partially miscible blends have been utilized for gas separation applications where their performance relies on membrane morphology, specific volume fraction, and the size and shape of the dispersed and continuous phase [99].

Table 6 summarizes several PI membranes’ performance by PI-based blends for H_2_/CO_2_ separation. PES and PSf have been introduced as potential blending polymers [76] as both polymers are miscible with PI, and they consist of a strong anti-plasticizing ability that enhances the CO_2_ plasticization resistance even at high feed pressure (>30 bar). Hamid et al. [61] proposed blending PSf with PI (P84) polymers to create a membrane suitable for H_2_/CO_2_ separation. The polymer blending process involves dissolving the equal ratios of PSf and P84 in the solvent mixture of N-methyl pyrrolidinone (NMP) and tetrahydrofuran (THF), followed by membrane synthesis using the phase inversion technique. This method is preferred for its simplicity and effectiveness in achieving the desired polymer combination. The findings showed that the PSf/PI-60 membrane achieved the highest permeability of H_2_ (348 GPU), with a selectivity of 4.4, higher than neat PSf (2.9), and P84 (3.1) for H_2_/CO_2_ [61]. The study also highlighted the importance of selecting the appropriate evaporation time, as it is significantly related to the formation of membrane structures. The 60 s of the evaporation process formed a slightly bigger pore size (~0.68 μm) when compared to a longer evaporation time (180 s) [61]. This could be explained by the stronger intermolecular forces between the polymer, resulting in a tighter, oriented, and denser structure with lesser free volume [101]. In addition, the thermal properties of the blended membrane exhibited a single T_g_ and showed an intermediate T_g_ value between neat PSf and the P84 membrane, which indicates good miscibility and compatibility that enhances the thermal stability of the blended membrane [61]. This thermal stability enhancement and performance highlights the effectiveness of the blending approach in improving gas separation efficiency.

**Figure 12:**
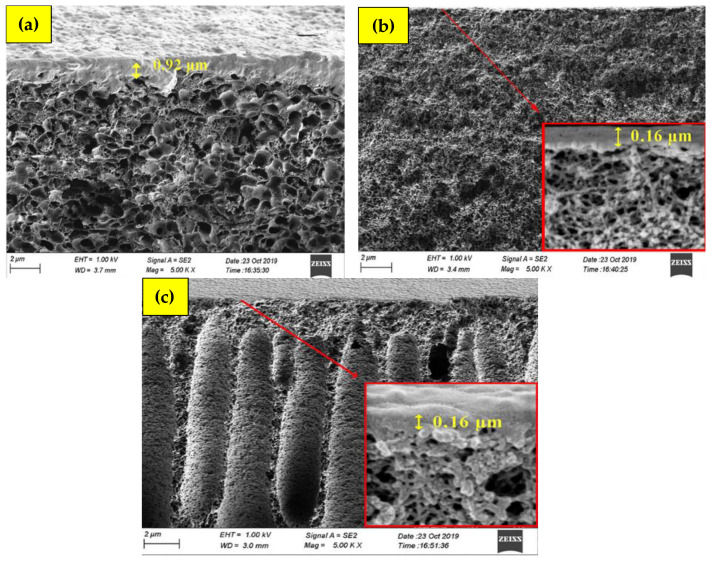
(**a**) PES/P84 membrane with ratios of 25:75; (**b**) PES/P84 membrane with ratios of 50:50; and (**c**) PES/P84 membrane with ratios of 75:25. (**a**,**b**) form a thick sponge-like structure, while (**c**) forms a finger-like sublayer structure. Reproduced with permission from [53], De Gruyter, 2021.

In another study, Nasir et al. [53] blended polyethersulfone (PES) with PI (P84), following the same method used by Hamid et al. [61] with the aim of enhancing H_2_/CO_2_ separation. Different composition ratios of PES and P84 were studied to optimize the H_2_/CO_2_ separation, and the findings show that the PES/P84 blend with ratios of 75/25 wt. % exhibited the highest H_2_ permeability (53.70), with roughly four-fold increases from the PES/P84 with the ratios of 25/75 wt. % (12.70). Moreover, this PES/P84 (75/25 wt. %) blended polymer shows the highest H_2_/CO_2_ selectivity (3.49) when compared with other blending ratios [53]. This may be contributed by the different membrane structures formed, where both PES/P84 polymers with low PES content form thick sponge-like structures, reducing the gas molecules to pass through, whereas blended PES/P84 with the highest PES content forms a finger-like sublayer in the membrane structure, facilitating the gas transport [102], as illustrated in Figure 12.

Hosseini et al. [103] blended Matrimid and polybenzimidazole (PBI) in various weight ratios. The findings confirmed that the blend of Matrimid/PBI formed a miscible blend, indicating a single T_g_ across different compositions through differential scanning calorimetry (DSC). This may be attributed to the strong hydrogen bonding interaction between the N-H group of PBI and the C=O group of the Matrimid polymer chain [104,105,106]. Thus, incorporating PBI is believed to enhance stiffness, restrict the segmental polymer chain’s mobility, and decrease the d-spacing between the polymer chains. This surely will affect the diffusivity selectivity of the gas molecule and stabilize the membrane structure, withstanding CO_2_ plasticization [103]. In terms of membrane performance, the presence of PBI content increases by about 1.5-fold and reaches the H_2_/CO_2_ selectivity up to 9.43 for membrane with a composition of 25:75 (Matrimid/PBI) [103].

In another study, Hosseini et al. [107] study the blending of a few PI types and PBI, as indicated in Table 3. H_2_/CO_2_ selectivity for neat polymers is higher than the blended polymers except for PBI/Matrimid, which increases the H_2_/CO_2_ selectivity from 3.88 to 6.08. The studies show that Matrimid is a better partner of PBI when compared with other PI types. The findings also show that H_2_ permeability for PBI/Matrimid decreases from 27.16 to 13.06 due to the strong interaction between the functional groups in both polymers. PBI content in the blend membranes significantly affects membrane properties. This is because the rigid aromatic chain in the PBI structure improved the discrimination between the kinetic diameter of H_2_ (2.89 Å) and CO_2_ (3.3 Å).

Huang [66] proposed the blending of 6FDA-DAM with PBI by dissolving in N-N-dimethylacetamide (DMAc), followed by stirring these solutions at 80 °C for 1 day, which led to the formation of complete dissolution and homogeneity of the polymer that offered an increase in H_2_/CO_2_ selectivity. However, the blend of these polymer materials forms an immiscible blend [66]. Due to this reason, a small amount of ZIF-8 nanoparticles is introduced into this polymer membrane to improve their compatibility, resulting in better membrane performance [66]. The presence of PBI in the blend membrane increases the H_2_/CO_2_ selectivity up to 15.2 because PBI has a higher affinity for H_2_ molecules attributed to its rigid structure, higher thermal stability, and selective permeation [66,108,109].

In a recent study by Kayadoe et al. [63], the study proposed the addition of low P84 in the fabrication of PSf/P84 blended membranes for H_2_/CO_2_ separation. The study used the same methods as proposed by Hamid et al. [61]. This study suggested that a high P84 content deteriorates the membrane’s mechanical properties. When a small amount of P84 was added to the PSf membrane (in ratios of 1:0.025 and 1:0.05), the membrane surface did not change much [63]. However, when the P84 content was added at a ratio of 1:10, the surface started to exhibit a rougher and more uneven surface with scattered pores [63]. Therefore, it will increase the porosity, resulting in an irregular finger-like region, leading to reduced membrane stability and mechanical strength [63]. This is because P84 has lower tensile strength than PSf [61,63]. Lower tensile strength led to more stiff and brittle polymer membrane materials [61]. This then decreases membrane flexibility and reduces the membrane properties and performance.

**Table 6:** Gas performance is based on several PI-based blend membranes (“na” represents unavailable data from literatures).

Description	T/P (°C/atm)	H_2_ Permeability (Barrer)	CO_2_ Permeability (Barrer)	H_2_/CO_2_ Permselectivity	Ref.
P84	na/1	233	76	3.1	[61]
P84/PSf	na/1	348	86	4.4	
PSf	na/1	139	39	3.6	
PES/P84 (25:75)	na	39.66	12.70	3.12	[53]
PES/P84 (50:50)	na	42.86	12.46	3.44	
PES/P84 (75/25)	na	187.30	53.70	3.49	
Matrimid	35/na	27.16	7.00	3.8	
Matrimid/PBI (75:25)	35/na	19.72	4.19	4.0	[103]
Matrimid/PBI (50:50)	35/na	13.06	2.16	6.0	
Matrimid/PBI (25:75)	35/na	5.47	0.58	9.4	
Torlon	35/na	4.44	0.83	5.3	[107]
PBI/Torlon (50:50)	35/na	3.75	0.62	6.0	
P84	35/na	9.09	1.37	6.6	
PBI/P84 (50:50)	35/na	6.88	1.60	4.3	
Matrimid	35/na	27.16	7.00	3.8	
PBI/Matrimid (50:50)	35/na	13.06	2.16	6.0	
PBI	35/3	1.37	0.05	27.4	[66]
6FDA-DAM	35/3	1020	9.43	1.1	
PBI/6FDA-DAM	35/3	6.76	0.45	15.2	
PSf/P84 (1:0.025)	30/2	3.0	na	6.25	[63]
PSf/P84 (1:0.05)	30/2	16.66	na	3.61	
PSf/P84 (1:0.10)	30/2	4.25	na	3.05	
PSf/P84 (1:0.20)	30/2	126.86	na	5.38	

### 4.2. Key Parameter in Blending Modification: Compatability

It must be noted that several key parameters must be considered to achieve better membrane features for H_2_ purification. The goal of H_2_ purification, using a blended polymer membrane, is to maximize H_2_ purification while maintaining membrane strength and durability. The key parameter for a successful polymer blend is the compatibility of the polymer pairs at the molecular level, which can be observed from the blending material selection and the polymer blending ratio.

#### 4.2.1. Blending Material Selection

Polymer material selection is essential in polymer blending modification. A good blending material pair can improve the PI membrane’s resistance to CO_2_ plasticization. The important factor for blending PI with other polymers is that it has very low permeability and plasticization resistance [99]. It is worth noting that every individual polymer has excellent properties. Taking this idea, therefore, blends the PI polymer with a polymer, exhibiting high CO_2_ plasticization resistance, which is relatively easy and a preferred method of improving the membrane properties [80]. Extensive research has proven that blending PI with PES, PSf, and other high-CO_2_ plasticization-resistant polymers can suppress CO_2_ plasticization, as discussed in Section 4.1. Thus, it relatively helps improve H_2_/CO_2_ selectivity by improving their mechanical and thermal properties. However, to date, blending modification cannot eliminate this CO_2_ plasticization completely. It can only reduce this effect at a certain level.

In addition, a miscible blend can offer uniform performance along with improved thermal and chemical properties [73]. Miscible blends are formed by mixing two or more polymers, resulting in a transparent and homogeneous (single-phase) solution. This can be observed physically and it also exhibits a single glass transition temperature (T_g_) when analyzed using characterization analysis [60]. In the field of H_2_ purification, ref. [107] reported that a miscible blend of Matrimid and PBI improves the performance of the neat PI membrane. This improvement occurs by increasing the packing density of the polymer chains and reducing their segmental mobility. As a result, these changes positively affect the diffusivity selectivity of the membrane with H_2_/CO_2_ selectivity up to 9.43 with a composition of 25:75 (Matrimid/PBI). Notably, PBI functioned to promote chain stiffness in the polymer chain, leading to a tighter and narrower structure of the polymer chain. Thus, it stabilizes membrane structure withstanding the CO_2_ plasticization effect, which reduces the H_2_ permeability and increases H_2_/CO_2_ selectivity.

#### 4.2.2. Polymer Blending Ratio

Polymer blending ratio or composition significantly affects the final membrane properties and structure. The optimal blending ratio of polymer is crucial in enhancing the modified membrane. Most studies, as depicted in Table 3, show that a good-performance PI membrane is directly related to a low PI ratio in the blended membrane if using other polymer types, such as PSf, PBI, and PES. PI weight determines the critical CO_2_ plasticization pressure of the blended membrane, as tabulated in Table 7.

It is believed that the high content of PI, specifically using P84, will induce significant changes in the membrane structure, including hydrophilicity, pore structure, and mechanical properties [63]. Higher PI content significantly deteriorates the mechanical properties of the blended polymer as it promotes brittleness on the membrane properties [63]. This is because the high content of PI in the membrane makes it the elongation values decrease and low tensile strength [61,63], increasing the rigidity of the membrane, which is unsuitable for using this membrane under high pressure and gas flow rates. This is because it will reduce the lifespan of the membrane due to the risk of membrane damage in a short period [63]. The low tensile strength may be attributed to the irregular finger-like structure and macrovoids observed in the sponge-like region of the blended membranes with high PI content. It should be noted that irregular structure leads to a decrease in stability and mechanical strength, suggesting that the use of PSf, for example, will help in reducing brittleness because it has a more flexible structure than P84 [61]. However, it should be noted that the presence of P84 promotes better thermal stability due to the high thermal decomposition (Td) that P84 has, even at the low content of 1:0.20 [63]. This indicates that blending PSf or PES with P84 is still preferable as a blending pair for membrane fabrication with a low P84 content.

**Figure 13:**
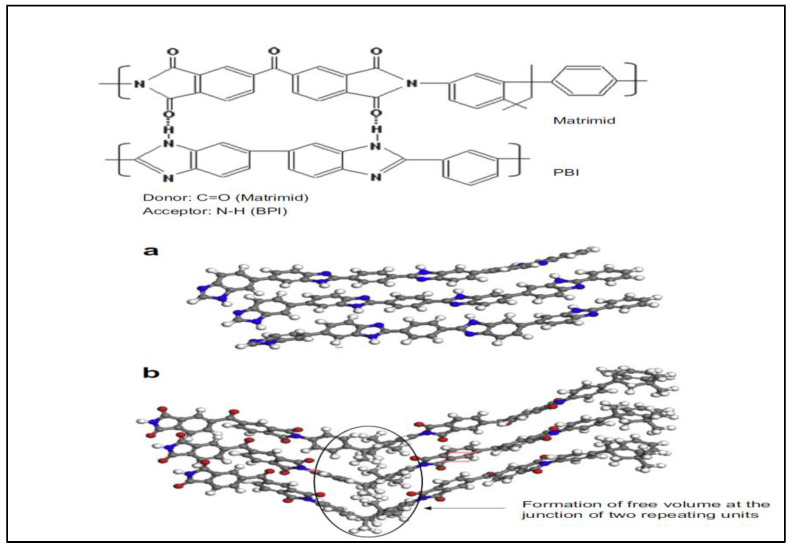
The formation of hydrogen bonds between Matrimid and PBI. (**a**) Molecular simulation of Matrimid structure and (**b**) molecular simulation of PBI structure. Reproduced with permission from [103], Elsevier, 2008.

When blending PI with PBI, specifically for the Matrimid membrane, it can also be seen that a low PI content is more suitable. Analyzing the Matrimid and PBI structures as illustrated in Figure 13, it can be seen that both structures are more or less similar in their backbone structure and repeating units, with different or fewer presences of side oxygen groups, as well as large methyl groups in the Matrimid structure, making the polymer chain hindered from coming closer together, larger FFV, making PBI has better chain packing than Matrimid [103]. This blending of PBI and Matrimid then improves the polymer chain packing by forming the hydrogen bonding between both polymers, making the polymer chain less flexible to move or rotate [103].

Inversely, for the blending of Matrimid/P84, a higher content of P84 in the blending process is required. A higher content of P84 in the polymer blend leads to higher resistance to the CO_2_ plasticization effect [80]. The ability of P84 to reduce CO_2_ sorption is mainly due to the alteration of the chain packing of the blended membrane. The higher the P84 content, the denser the chain packing of the blended membrane, and the lower the d-spacing, which then reduces the CO_2_ sorption [80]. Thus, the polymer blending ratio of the blending pair depends on the type of polymer material used for the blending modification because every material has its own unique structures.

## 5. Synergizing Polymer Blending and Crosslinking Modification for H_2_ Purification

Synergizing polymer blending and crosslinking modification on PI neat membranes is an advanced and promising method designed to significantly enhance the perm-selectivity of H_2_/CO_2_ separation while ensuring membrane stability under longer operation. By optimizing the interactions between the different polymer components and crosslinker, this synergy modification gives new membrane features that enhance membrane properties, facilitating better separation without compromising durability.

**Figure 14:**
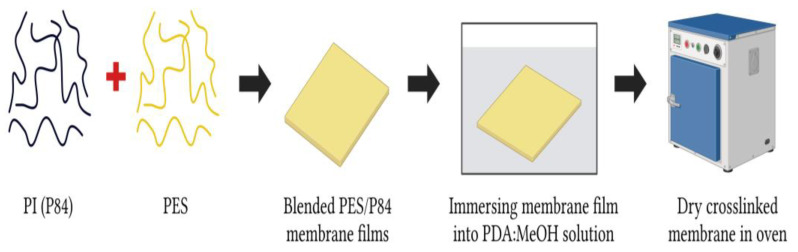
The modification strategy by crosslinked the blended PI/PES membrane.

In 2021, Nasir et al. [53] employed a P84/PES blend membrane followed by diamino crosslinking for H_2_/CO_2_ separation. Surprisingly, the modified PI with a composition of 75/25 wt. % (PES/P84) and 15 min-crosslinked membrane improved H_2_/CO_2_ selectivity up to 88.9%, surpassing Robeson’s upper bound curve for H_2_/CO_2_ gas pair performance. The study was conducted by immersing the blended PES/P84 membrane films into the PDA solution at room temperature. The details of the modification strategy as illustrated in Figure 14. The membrane was thoroughly washed with fresh methanol to remove any residual chemicals and dried in the oven before use.

**Figure 15:**
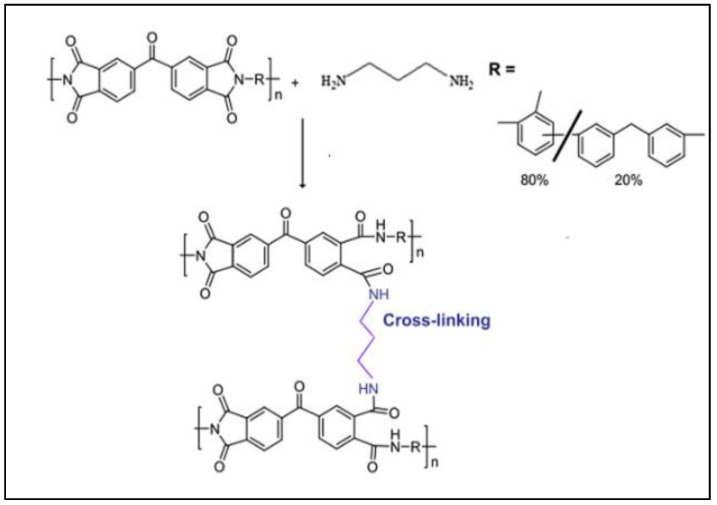
The possible reaction mechanism between imide group from P84 and PDA. Reproduced with permission from [53], De Gruyter, 2021.

It can be seen that the polymer blend of this modified membrane improved both H_2_ permeability and H_2_/CO_2_ selectivity of the neat membrane, whereby the diamine crosslinking hindered the CO_2_ permeability, which makes better H_2_/CO_2_ separation. The modification decreases the d-spacing of the membrane from 5.04 Å to 4.9 Å. This is because the diamine changes the packing formation of the polymer chain, tightening the membrane microstructures and resulting in improved H_2_/CO_2_ separation performance. As mentioned above, the optimal crosslinking was 15 min, where longer crosslinking time decreased the separation performance due to the opening of imide rings and formation of amide groups, which reduced the polymer backbone rigidity, caused the polymer chain to be flexible, and reduced the H_2_/CO_2_ selectivity (Figure 15) [53].

In addition, the blended membrane performance improved significantly after the polymer blending modification upon 15 min, which is from 3.49 (blended PES/P84 with an optimized blended composition of 75:25) to 6.87 (crosslinked PES/P84). Surprisingly, the H_2_ permeability is only slightly affected, which is from 187.30 (blended PES/P84) to 105.60 (crosslinked PES/P84), and the CO_2_ permeability decreases significantly, which is from 53.70 (blended PES/P84) to 15.36 (crosslinked PES/P84). However, this study was conducted under controlled conditions and only evaluated through a pure gas permeation test. This emphasizes the need for further exploration to assess performance in real-world situations accurately, and it remains to be determined whether this synergistic modification truly enhances both the permeability and selectivity of H_2_/CO_2_ separation. Therefore, a fundamental investigation into these modifications is recommended to provide a comprehensive understanding of how this effect can enhance H_2_ selectivity and membrane durability in the H_2_/CO_2_ separation process.

In another study by Hosseini et al. [103] also shows that combining the blending and crosslinking modification strategy significantly improved the crosslinked-blended PI membrane performance for H_2_/CO_2_ separation. The study was conducted by blending the PI (Matrimid) with poly[2,20-(1,3-phenylene)-5,50-bibenzimidazole (PBI) in NMP solution and crosslinked it with different types of diamine (p-xylene diamine and p-xylene chloride) in methanol solution. The study observed that the presence of PBI content enhances the membrane performance by increasing the selectivity due to an increase in chain packing density which, as a result, reduces the segmental motion of the polymer chain. The blended Matrimid/PBI with a composition ratio of 25:75 crosslinked with p-xylene diamine shows the best H_2_/CO_2_ selectivity of 26.09. The study highlights that p-xylene diamine significantly gives an impact to the membrane performance than that of p-xylene dichloride. This is because p-xylene diamine has a higher tendency for crosslinking reaction than p-xylene chloride. The comparison between both studies is depicted in Table 8.

To summarize, the crosslinking and polymer blending process in the membrane system alters the membrane’s properties. In most cases, crosslinked modification enhances the total performance of the PI membrane. In general, the crosslinking reaction will increase the H_2_ selectivity and decrease the H_2_ permeability as it tightens the interstitial d-spacing in the polymer network. Besides, polymer blending will improve the H_2_ permeability and enhance membrane durability against CO_2_ plasticization.

Table 9 summarizes the most promoted characterizations after blending and crosslinking modifications. Even though the temperature usually affects the reaction in any process, we are not discussing it here as the high temperature is not significant for big-scale industry as it will increase the cost, and the separation can operate under room temperature.

## 6. Conclusions and Future Outlooks

Researchers have progressively developed and improvised diamine-crosslinked and blended PI membranes as they significantly have the potential to improve the neat PI membrane for H_2_ purification. Its utilization can improve energy generation efficiency and mitigate the adverse effects on the environment. This article thoroughly reviews how crosslinking reactions and polymer blending can improve H_2_ purification using this modified PI membrane. It purifies H_2_ from the mixed gases better than the neat PI membrane.

However, some limitations exist in developing this modified polymer membrane for commercialization. More effort is necessary to develop a high-performance PI membrane for H_2_ purification. The future outlooks for the PI membrane are as follows:

1. Enhancing Gas Separation Performance—The optimal blending ratio of the polymer pairs is critical for blended PI membranes as it significantly affects the membrane properties. A higher content of polymer that has higher CO_2_ plasticization in the blended membranes enhances the membrane to suppress CO_2_ plasticization when compared with a neat PI membrane. This is crucial to mitigate the challenge that neat PI suffers as it easily swells due to CO_2_ plasticization, reducing the membrane performance in purifying H_2_ gases. High-stability membranes prevent plasticization caused by CO_2_ gases and maintain their separation in the long run to ensure consistent permeation rates and selectivity, as well as reduce replacement costs and maintenance. In addition, for crosslinked PI membranes, it is significantly proved that the modified membrane can improve the H_2_ selectivity and surpass the upper bound curve in the Robeson plot, even slightly decreasing the H_2_ permeability. Optimizing the crosslinking parameters ensures that the developed modified PI membranes achieve at least high H_2_ selectivity while at least maintaining the H_2_ permeability to achieve a more efficient separation process. It is important to understand that selectivity indicates “quality,” whereas permeability refers to “quantity”. Therefore, the right balance of crosslinker concentration and reaction time is important to ensure the optimal chain packing of the polymer chain that sufficiently allows only H_2_ to permeate. Nevertheless, the right choice of crosslinker types that enable the crosslinker to diffuse into the polymer chain effectively is the most important parameter for a complete crosslinking reaction. A diamine crosslinker type that offers stronger interactions with polymer chains and better control over chain packing and FFV is preferable for optimizing the membrane performance. In addition, exploring crosslinker solution types may be significantly important to study the effectiveness of crosslinking reactions.
2. Advancing fundamental studies on crosslinking reaction in modified PI membranes—For crosslinking modification, studying the kinetics of diamine crosslinking helps to determine how crosslinking occurs at the molecular level, including their reaction pathway and activation energy. Studying the interaction of the crosslinker and the polymer chain provides insight into how it influences the bonding strength between the crosslinker and the polymer chain. Therefore, the synergy between fundamental studies and optimization of crosslinking parameters ensures a data-driven approach to refine the crosslinking strategy, leading to a developed membrane with superior performance and desired properties.

In summary, H_2_ purification by crosslinked and blended polymer membranes significantly improved desired H_2_ selectivity and membrane durability. However, it will reduce the permeability of H_2_ gas. With the continuous improvement of developed membrane materials, gas separation membrane technology will continue to progress, providing better solutions to address energy and environmental issues and benefiting all industries. It is recommended that membrane fabricators, researchers, and investors engage in collective discussions to share their expertise. This collaboration will drive the advancement of membrane technology, ensuring it meets industry standards while pursuing common objectives.

## Figures and Tables

**Table 1:** Advantages and disadvantages of several types of H_2_ purification technologies [32,33,34].

Technology	Principles	Advantages	Disadvantages
Cryogenic distillation	Separate H_2_ from mixed gases under high-pressure and low-temperature conditions	Suitable for applications that need H_2_ purity up to 95% No chemical usage	Use high energy consumption Use larger and more complicated equipment (compressor and cooling system) Complicated operation due to heat exchangers’ sensitivity Need to remove contaminant gases before purifying H_2_ gas High risk of equipment blockage if there is the presence of contaminant gases High capital and operational cost
Pressure swing adsorption	Use solid adsorbents to adsorb contaminant gases and produce high purity of H_2_ under high pressure	Produce high purity of H_2_ up to 99.999% Operated at ambient temperature	High capital and operational cost Complicated operation
Membrane separation	Use a selective barrier that allows H_2_ to permeate from the mixed gases	Flexible and simple operation Suitable for low-temperature applications Low carbon footprints Compact equipment Low energy usage Easy for installation and integration with industrial processes	Only suitable for small-scale application Low H_2_ purity due to trade-off limit between permeability and selectivity Low durability, which causes a short lifespan High capital and operational cost
Chemical absorption	Use chemical absorbent to absorb impurities and allow H_2_ to pass through in the gas phase from the absorbent	Operated at low pressure Use low energy H_2_ purity in the range of 95–97%	Use a high volume of solvent Some of the solvents are corrosive

**Table 2:** H_2_/CO_2_ selectivity for commercially available polymer materials [52].

Polymer Material Types	H_2_/CO_2_ Selectivity
Cellulose acetate (Separex)	2.4
Polysulfone (Permea)	2.5
Polyimide (Ube)	3.8
Tetrabomopolycarbonate (MG)	3.5
Silicone rubber (Silar)	0.2

**Table 3:** The comparison of H_2_/CO_2_ selectivity between pure PI and modified-PI membrane (“na” represents unavailable data from literatures).

Descriptions	T/P (°C/atm)	H_2_ Permeability (Barrer)	CO_2_ Permeability (Barrer)	H_2_/CO_2_ Selectivity	Ref.
6FDA-durene	35/3.5	na	na	na	[86]
DAB-AM-4-modified 6-FDA-durene (60 min)	35/3.5	na	na	3.7	
DAB-AM-8-modified 6-FDA-durene (60 min)	35/3.5	na	na	na	
DAB-AM-16-modified 6-FDA-durene (60 min)	35/3.5	na	na	na	
P84	na-/3	132.5	25.8	5.11	[65]
P84	na/4	151.6	28.1	5.40	
P84	na/5	191.2	35.0	5.31	
BuDA-modified P84 (10 min)	na/3	778.1	193.0	4.03	
BuDA-modified P84 (10 min)	na/4	804.1	175.6	4.58	
BuDA-modified P84 (10 min)	na/5	804.1	206.8	3.90	
BuDA-modified P84 (30 min)	na/3	594.8	101.0	5.88	
BuDA-modified P84 (30 min)	na/4	602.7	103.5	5.82	
BuDA-modified P84 (30 min)	na/5	654.3	107.3	6.09	
6FDA-durene	35/7	~600	na	~1	[44]
PDA-modified 6FDA-durene (1 min)	35/7	~250	na	~4	
PDA-modified 6FDA-durene (5 min)	35/7	~20	na	~40	
PDA-modified 6FDA-durene (10 min)	35/7	~15	na	101	
BuDA-modified 6FDA-durene (5 min)	35/7	~18	na	~38	
EDA-modified 6FDA-durene (5 min)	35/7	~150	na	~5	
P84	25/1	18.4	3.84	4.78	[87]
5 wt. % DAMP-modified P84	25/1	1.84	0.205	9.00	
10 wt. % DAMP-modified P84	25/1	1.10	0.087	12.64	
6FDA-durene	na/3.5	600	581	1.03	[85]
EDA-modified 6FDA-durene (5 min)	na/3.5	73.4	1.97	37.3	
EDA-modified 6FDA-durene (10 min)	na/3.5	32.6	0.32	102	
P84	na/3	na	na	4.3	[64]
(1:0) PDA:BUDA-modified 15 wt. % P84 (5 min)	na/3	na	na	7.3	
(0.8:0.2) PDA:BUDA-modified 15 wt. % P84 (5 min)	na/3	na	na	7.84	
(0.6:0.4) PDA:BUDA-modified 15 wt. % P84 (5 min)	na/3	na	na	4.6	
(0.4:0.6) PDA:BUDA-modified 15 wt. % P84 (5 min)	na/3	na	na	4.5	
(0.2:0.8) PDA:BUDA-modified 15 wt. % P84 (5 min)	na/3	na	na	1.86	
(0:1) PDA:BUDA-modified 15 wt. % P84 (5 min)	na/3	na	na	0.72	
6FDA-ODA/NDA	35/3.5	69.78	29.01	2.3	[84]
EDA-modified 6FDA-ODA/NDA (15 min)	35/3.5	51.50	9.47	5.4	
EDA-modified 6FDA-ODA/NDA (30 min)	35/3.5	36.84	2.08	17.7	
EDA-modified 6FDA-ODA/NDA (60 min)	35/3.5	26.72	0.98	27.1	
EDA-modified 6FDA-ODA/NDA (90 min)	35/3.5	22.70	0.76	29.7	
EDA-modified 6FDA-ODA/NDA (120 min)	35/3.5	16.55	0.71	23.4	
PDA-modified 6FDA-ODA/NDA (15 min)	35/3.5	60.14	9.06	6.6	
PDA-modified 6FDA-ODA/NDA (30 min)	35/3.5	36.55	1.58	23.2	
PDA-modified 6FDA-ODA/NDA (60 min)	35/3.5	23.34	0.60	39.2	
PDA-modified 6FDA-ODA/NDA (90 min)	35/3.5	16.48	0.26	64.1	
PDA-modified 6FDA-ODA/NDA (120 min)	35/3.5	13.90	0.23	60.0	
BuDA-modified 6FDA-ODA/NDA (15 min)	35/3.5	70.74	14.18	5.0	
BuDA-modified 6FDA-ODA/NDA (30 min)	35/3.5	63.04	13.33	4.7	
BuDA-modified 6FDA-ODA/NDA (60 min)	35/3.5	55.11	6.24	8.8	
BuDA-modified 6FDA-ODA/NDA (90 min)	35/3.5	45.82	3.98	11.5	
BuDA-modified 6FDA-ODA/NDA (120 min)	35/3.5	38.55	1.84	20.9	
DAP-modified 6FDA-6FPA	35/1	8.264	2.873	2.88	[88]
17% *v*/*v* DAP-modified 6FDA-6FPA (65 min)	35/1	29.318	6.602	4.44	
10% *v*/*v* DAP-modified 6FDA-6FPA (10 min)	35/1	9.953	3.479	2.86	
3% *v*/*v* DAP-modified 6FDA-6FPA (120 min)	35/1	7.354	0.988	7.44	

**Table 4:** The comparison characteristics between EDA, PDA, and BuDA [84].

Diamine Crosslinker Types	Nucleophilicity	Molecular Dimension	Effectiveness for Crosslinking Reaction	Findings
EDA	Most nucleophilic	5.5 Å (Smallest)	Less effective	Highly severe chemical etching and significant chemical grafting were unable to hinder the chain-to-chain movement. Main-chain scissions are the most severe.
PDA	Moderate nucleophilic	6.7 Å (Moderate)	Most effective	Highest degree of crosslinking reaction promotes chain rigidity
BuDA	Least nucleophilic	8.0 Å (Largest)	Less effective	Presence of chemical grafting and severe chemical etching Chain is still able to mobile as the molecule backbone is more flexible than EDA and PDA.

**Table 5:** The comparison of crosslinking modification methods.

Modification Method	Descriptions	Advantages	Limitation	Ref.
Post-synthesis	Membrane is immersed in the crosslinker solution after it has solidified.	Room temperature conditions Simple and easy method	Uneven membrane thickness of the top layer Use a large volume of solvent for the immersion method Difficulty in controlling pore size of membrane surface Membrane swelling Lower gas permeability and selectivity than the vapor phase method Deteriorate mechanical properties of membrane	[86]
Vapor phase	Modify the outer layer of the membrane without modifying the internal membrane structure	Improve tensile properties Environmental-friendly Higher permeability and selectivity of gas separation than the solution-modified method Use less chemical Minimal swelling process Reduce cost Suitable more for hollow fiber membrane	Sensitive to humidity Complicated method Difficulty in controlling temperature for the modification Use high temperature and energy usage	[85,93]

**Table 7:** The critical CO_2_ pressure of PSf/PI blend membranes [76].

PI/PSf (% *w*/*w*)	CO_2_ Plasticization Pressure (atm)
0/100	>50
20/80	>35
50/50	~30
80/20	18
100/0	15

**Table 8:** The comparison of membrane performance between unmodified and modified PI membranes with combined blending and crosslinking modification strategies (“na” represents unavailable data from literatures.

Descriptions	T/P (°C/atm)	H_2_ Permeability (Barrer)	CO_2_ Permeability (Barrer)	H_2_/CO_2_ Selectivity	Ref.
P84	na	187.3	53.7	3.49	[53]
PDA-modified PES/P84 (75:25) (5 min)	na	288.48	76.12	3.79	
PDA-modified PES/P84 (75:25) (10 min)	na	310.78	70.16	4.43	
PDA-modified PES/P84 (75:25) (15 min)	na	105.60	15.36	6.87	
PDA-modified PES/P84 (75:25) (30 min)	na	122.43	30.90	3.96	
Matrimid	35/3.5	27.16	7.00	3.88	[103]
Matrimid/PBI (25:75)	35/3.5	5.47	0.580	9.43	
p-xylenediamine-modified Matrimid/PBI (25:75) (5 days)	35/3.5	4.09	0.209	19.56	
p-xylenediamine-modified Matrimid/PBI (25:75) (10 days)	35/3.5	3.60	0.138	26.09	
p-xylene chloride-modified Matrimid/PBI (25:75) (5 days)	35/3.5	5.34	0.453	11.79	
p-xylene chloride-modified Matrimid/PBI (25:75) (10 days)	35/3.5	4.04	0.306	13.02	

**Table 9:** The most significant modification changes after the crosslinking and polymer blending process.

Parameters	Changes After Modifications
Crosslinking time	Reduce membrane pore size with increasing time Tighten interstitial d-spacing of membrane Increase the degree of crosslinking until optimal crosslinking time
Crosslinker types	Molecular dimension and nucleophilicity of molecules affect the crosslinking reaction Weaker nucleophile crosslinker difficult to diffuse in the polymer chain network Large molecular dimension of crosslinker incomplete crosslink with polymer chain
Crosslinker concentration	Increasing crosslinker concentration leading to decrease the H_2_ permeability and increasing H_2_ selectivity Optimal crosslinker concentration for sufficient crosslinking reaction
Crosslinking modification method	Depends on the shape of fabricated membrane Vapor phase method more suitable for hollow fiber membrane but use complicated method Post-synthesis method well-known to be used for flat sheet membrane
Crosslinker solution types	Methanol is well-known to swell the membrane, facilitating the crosslinking reaction to occur
Polymer blending compositions	High content of high resistance polymer is preferable to increase CO_2_ plasticization resistance
Polymer materials selection	PI is preferable to blend with polymers that have higher CO_2_ plasticization resistance
Ratios of crosslinker to the blended polymers	Need to adjust as too high crosslinker concentration will decrease H_2_ selectivity

## Abbreviations

The following abbreviations are used in this manuscript:

AC	acetate cellulose
BuDA	1,4-diaminobutane
CF_3_	trifluoromethyl
CHP	combined-heat power
CO	carbon monoxide
CO_2_	carbon dioxide
DAB	diaminobutane
DAMP	1,5-diamino-2-methylpentane
DAP	1,5-pentadiamine
DMAc	N-N-dimethylacetamide
DMF	dimethylformamide
DMSO	dimethyl sulfoxide
EDA	ethylenediamine
FFV	fractional free volume
GHG	greenhouse gas
HFC	hydrogen fuel cell
HFCV	hydrogen fuel cell vehicle
H_2_	hydrogen
H_2_O	water
H_2_S	hydrogen sulphide
NG	natural gas
NMP	N-methyl pyrrolidinone
PBI	poly[2,20-(1,3-phenylene)-5,50-bibenzimidazole
PC	polycarbonate
PDA	1,3-diaminopropane
Pd	palladium
PI	polyimide
PSf	polysulfone
P2G	Power-to-Gas
THF	tetrahydrofuran
T_g_	glass transition temperature
